# A Computational Approach for the Prediction of Treatment History and the Effectiveness or Failure of Antiretroviral Therapy

**DOI:** 10.3390/ijms21030748

**Published:** 2020-01-23

**Authors:** Olga Tarasova, Nadezhda Biziukova, Dmitry Kireev, Alexey Lagunin, Sergey Ivanov, Dmitry Filimonov, Vladimir Poroikov

**Affiliations:** 1 Department of Bioinformatics, Institute of Biomedical Chemistry, 119121 Moscow, Russia; nad.smol@gmail.com (N.B.); alexey.lagunin@ibmc.msk.ru (A.L.); smivanov7@gmail.com (S.I.); dmitry.filimonov@ibmc.msk.ru (D.F.); vladimir.poroikov@ibmc.msk.ru (V.P.); 2 Central Research Institute of Epidemiology, 111123 Moscow, Russia; dmitkireev@yandex.ru; 3 Department of Bioinformatics, Pirogov Russian National Research Medical University, 117997 Moscow, Russia

**Keywords:** human immunodeficiency virus Type 1, HIV-1, treatment history, therapy failure, protease, reverse transcriptase, PASS, random forest

## Abstract

Human Immunodeficiency Virus Type 1 (HIV-1) infection is associated with high mortality if no therapy is provided. Currently, the treatment of an HIV-1 positive patient requires that several drugs should be taken simultaneously. The resistance of the virus to an antiretroviral drug may lead to treatment failure. Our approach focuses on predicting the exposure of a particular viral variant to an antiretroviral drug or drug combination. It also aims at the prediction of drug treatment success or failure. We utilized nucleotide sequences of HIV-1 encoding protease and reverse transcriptase to perform such types of prediction. The PASS (Prediction of Activity Spectra for Substances) algorithm based on the naive Bayesian classifier was used to make a prediction. We calculated the probability of whether a sequence belonged (P_1_) or did not belong (P_0_) to the class associated with exposure of the viral sequence to the set of drugs that can be associated with resistance to the set of drugs. The accuracy calculated as the average Area Under the ROC (Receiver Operating Characteristic) Curve (AUC/ROC) for classifying exposure of the sequence to the HIV-1 protease inhibitors was 0.81 (±0.07), and for HIV-1 reverse transcriptase, it was 0.83 (±0.07). To predict cases of treatment effectiveness or failure, we used P_1_ and P_0_ values, obtained in PASS, along with the binary vector constructed based on short nucleotide descriptors and the applied random forest classifier. Average AUC/ROC prediction accuracy for the prediction of treatment effectiveness or failure for the combinations of HIV-1 protease inhibitors was 0.82 (±0.06) and of HIV-1 reverse transcriptase was 0.76 (±0.09).

## 1. Introduction

Human Immunodeficiency Virus Type 1 (HIV-1) causes acquired immunodeficiency syndrome (HIV/AIDS), a disease with severe complications, leading to death if no drugs are administered [1,2]. A high velocity of replication and a high rate of errors appearing during the replication characterize HIV-1. These two factors lead to a high mutation rate of HIV-1 [3]. Currently, the role of multiple HIV-1 proteins in HIV-1/AIDS disease pathogenesis and progression is under investigation, including the role of its structural proteins, as well as HIV-1 trans-activator of transcription (tat) protein [4,5]. These studies are essential because they might have an impact on the development of HIV-1 vaccines and novel therapeutic approaches (such as, for instance, “block-and-lock strategies”). Along with new strategies of HIV-1/AIDS treatment, antiretroviral therapy still is an effective method that allows for the reduction of the number of viral copies [6]. Combinations of antiretroviral drugs (antiretroviral therapy (ART)) are used to control HIV/AIDS infection [6]. ART combinations are based on the inhibitors of all three structural enzymes of HIV-1: Reverse Transcriptase (RT), Protease (PR), and Integrase (IN). Experimental tests allow for the evaluation of HIV-1 resistance against RT and PR inhibitors [7,8]. Several machine learning approaches predict the resistance and/or exposure of a particular HIV-1 variant to a drug on the basis of the nucleotide or amino acid sequences of the HIV-1 PR and RT [9,10,11,12,13,14,15,16,17]. Earlier, we reported computational approaches for predicting HIV-1 resistance to RT and PR inhibitors [10,18,19] based on sequences of HIV-1 variants collected from around the world, available from the Stanford HIV Resistance Database (STDB) [20]. We also showed that for predicting resistance against certain HIV variants, the usage of nucleotide sequences is preferable to amino acid sequences. The aim of the approach presented here is to predict associations between viral genotype and HIV-1 treatment history (sequence exposure to a drug) using the Bayes based PASS approach. The PASS approach is capable of predicting over 5000 types of biological activities, including pharmacological effects, mechanisms of action, toxic and adverse effects, interaction with metabolic enzymes and transporters, influence on gene expression, etc. The prediction of biological activity is based on the structural formula of the chemical compound. As we showed earlier, PASS can be applied to predict the resistance to HIV RT inhibitors on the basis of so-called position specific descriptors [19]. We focused on determining potentially useful combinations of drugs and those that may fail to display any remarkable therapeutic effect in the treatment of patients with a particular HIV-1 variant. All these steps are necessary to save time and effort in HIV-1 sequencing and resistance testing. Moreover, this approach allows users to take into account multiple mutations related to resistance towards a particular drug combination.

## 2. Results

We grouped HIV PR nucleotide sequences according to the following class types: (a) belonging to a viral variant exposed to certain drugs (treatment history, sequence exposure to a set of drugs)–“HIV PR treatment history dataset” and (b) the effectiveness of therapy by a combination of drugs–“The HIV PR combination dataset”.

In our approach, we assumed that there was an association between the drugs that were prescribed and taken by the patient and changes in the nucleotide sequences of HIV-1 encoding viral proteins. In Classification Type (a), the set of the drugs taken by a patient was considered as a particular class regardless of whether they were taken sequentially or simultaneously. In Type (b), only combinations simultaneously taken by a patient were considered to be a class.

### 2.1. Results of PASS Based Prediction of the Associations between Viral Genotype and Drug Set to which the Virus Was Exposed

The HIV PR treatment history dataset was used to predict associations between viral genotype and a set of drugs to which the virus was exposed. The prediction accuracy was identified through leave-one-out cross-validation (LOO CV) and fivefold CV. The corresponding AUC/ROC (Area Under ROC curve) values are given in Table 1. The prediction of associations between viral genotype and treatment history was obtained using the Bayes based PASS approach.

Table 1 shows that our approach could predict the association between a particular sequence and the set of antiretroviral drugs (treatment history) with an average AUC/ROC accuracy of 0.81. Our results for sequence classification according to a particular set of drugs taken by a patient either consequentially or simultaneously were similar or insignificantly exceeded those reported earlier [17] for datasets collected from the EuResist project (AUC/ROC 0.78).

**Figure 1:**
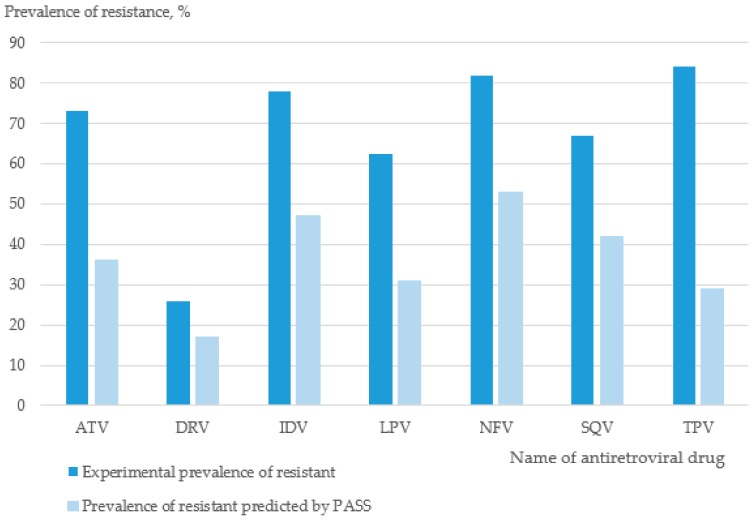
Prevalence of resistant samples among isolates (i) exposed to the drug (dark blue) and (ii) exposed to the drug according to Prediction of Activity Spectra for Substances (PASS) prediction (light blue). The values of prevalence were calculated from the HIV PR treatment history dataset and are associated with resistance to PR inhibitors. The HIV PR treatment history dataset was combined with resistance data. The isolate proportion was calculated as the number of drug resistant isolates divided by the total number of times the drug appeared in the treatment history. ATV, Atazanavir; APV, Amprenavir; DRV, Darunavir; FPV, Fosamprenavir; IDV, Indinavir; LPV, Lopinavir; NFV, Nelfinavir; RTV, Ritonavir, SQV, Saquinavir; TPV, Tipranavir.

We assumed that some mutations may occur in viral genes, resulting in higher viral fitness compared to wild-type HIV-1 when the virus is exposed to a particular drug (i.e., if a patient with a prevalent viral variant is taking an antiretroviral drug or drug combination). To check this hypothesis, we performed two computational experiments. First, we calculated the total number of isolates resistant to the drug in sets of sequences exposed to the same drug (i.e., included in the particular set of drugs taken by a patient). Second, we calculated the Positive Predictive Value (PPV), the number of sequences exposed to the drug that displayed resistance to the same drug in the experiment testing (according to STDB) and were predicted to be exposed to the same drug by PASS in an LOO CV procedure. The results are provided in Figure 1.

There was an association between the prevalence of resistant samples among isolates (i) exposed to the drug according to STDB and (ii) exposed to the drug according to PASS prediction (Pearson correlation coefficient between Sets (i) and (ii), *r* = 0.735). Therefore, if exposure of a particular isolate was predicted by PASS to an antiretroviral drug, one could assume that this isolate could be resistant to that drug with a certain probability. Therefore, prediction of treatment history could be regarded as an additional method in the computational approach developed for the optimization of antiretroviral therapy, but it could not be the only method.

### 2.2. Results of Predicting Association between Nucleotide Sequence, Clinical Parameters, and Immunological Effectiveness/Failure

The prediction of the effectiveness or failure of any treatment is based on the set of antiretroviral drug combinations taken by a patient and data on the sequencing of isolates collected from the patient’s blood plasma. The HIV PR combination dataset was used for prediction. For a prediction of treatment effectiveness/failure, we used the dataset of Treatment Change Episodes (TCE) from the STDB. Each file describing one TCE contained information about combinations of PR and RT inhibitors taken by a patient, clinical data on CD4^+^ cell number and viral RNA copies, nucleotide sequences encoding PR and RT, and the date when the sequence and clinical data were collected. Since nucleotide sequences in TCE are separately provided for PR inhibitors and RT inhibitors, we used information on PR sequences and PR inhibitors to build models for the viral effectiveness/treatment of PR inhibitors and performed the same for RT inhibitors. However, each TCE included PR inhibitors in combination with RT inhibitors; therefore, each patient took PR inhibitors along with RT inhibitors.

The PASS approach [21,22,23,24] was applied in combination with a random forest (RF) classifier to obtain P_1_ and P_0_ values reflecting the probability that a particular combination was associated with either therapeutic success or failure affecting the particular viral variant. P_1_ and P_0_ values, calculated by PASS in leave-one-out cross-validation, the number of CD4^+^ cells, and the number of copies of viral RNA were used as descriptors, as described in the Materials and Methods. Two types of antiretroviral therapy failure are considered in the literature [25]. According to the World Health Organization (WHO), immunological failure is associated with a persistent number of CD4^+^ cells damaged by HIV-1 that do not exceed 250 cells per mm^3^ followed by clinical symptoms or below 100 cells in mm^3^ regardless of any changes in the clinical status of the HIV-1 patient. Virological failure of therapy occurs when the ART combination fails to suppress a patient’s viral load to fewer than 1000 copies of RNA per 1 mL. The prediction results of immunological treatment effectiveness/failure are provided in Table 2.

Table 2 displays good prediction results for only several drug combinations; some are labeled as failed. We carefully analyzed the structure of the dataset and found some rarely appearing combinations, including Amprenavir (APV), Lopinavir (LPV, both failed and effective), Indinavir (IDV), LPV (failed), IDV, and Ritonavir (RTV) (failed). Therefore, prediction accuracy may be improved by increasing the number of data points in the dataset.

**Figure 2:**
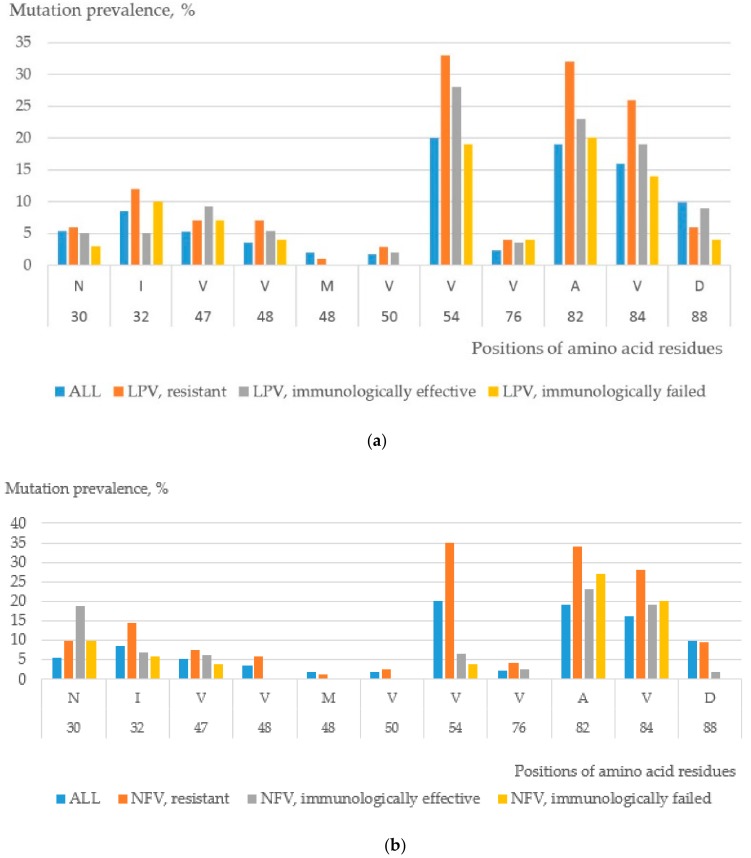
Distribution of amino acid mutations in HIV-1 protease for the whole set of isolates, a subset of resistant isolates, and isolates for which antiretroviral therapy was characterized as immunologically effective and failed for (**a**) LPV and (**b**) NFV. One letter codes with positions of the major drug resistance mutations are shown on the horizontal axis. N, asparagine; I, Isoleucine; V, Valine; M, Methionine; A, Alanine; and D, aspartic acid.

For illustrative purposes, we investigated the frequency of mutations in the major drug resistance positions for PR inhibitors that were obtained from the STDB. We calculated the frequency of mutations for the subset of amino acid sequences of (i) resistant isolates and (ii) isolates selected as being associated with the treatment effectiveness/failure of a particular drug. The frequency distributions of a single amino acid substitution are given in Figure 2 for the most representative datasets: Nelfinavir (NFV) and Lopinavir (LPV), immunological effectiveness/failure. The distributions for all drugs and drug combinations are provided in the Supplementary Materials (Tables S1 and S2). We suggest that a machine learning approach recognized differences of mutation prevalence between two groups (effective treatment/failure of treatment) because some minor mutations were taken into account since short nucleotide sequences had been used as descriptors. Therefore, each short nucleotide sequence may have contained both major and minor mutations that were distinguished on the basis of supervised learning.

The average AUC/ROC and AUC/ROC_20_ accuracy of the prediction of virological treatment effectiveness/failure for the combinations of HIV-1 PR inhibitors were 0.82 and 0.81, respectively. We provide detailed information about the combinations of drugs and corresponding accuracy in the Supplementary Materials (Table S3).

Building models using an RF classifier based on the calculation of P_1_ and P_0_ values and binary descriptors gave better average prediction performance compared to that of models built separately using either PASS or RF (AUC/ROC was 0.71 and 0.78, respectively, for immunological effectiveness/failure and 0.67 and 0.74 for virological effectiveness/failure of the therapy). Therefore, the combined application of RF models with probabilities calculated by PASS allowed for better recognition of the association between nucleotide sequence, clinical parameters, and immunological effectiveness/failure.

We reproduced the experiments on the prediction of drug exposure and treatment effectiveness/failure for HIV-1 RT. Average AUC/ROC and AUC/ROC_20_ values for the prediction of drug exposure were 0.828 and 0.80, respectively. The same values for the prediction of immunological effectiveness/failure of therapy were 0.71 (±0.01) and 0.70 (±0.01), respectively, and for virological effectiveness/failure of ART were 0.81 (±0.04) and 0.79 (±0.04), respectively. We provide detailed information about these accuracies in the Supplementary Materials (Table S4 for predicting sequence exposure to the drug and Tables S5 and S6 for predicting therapy effectiveness/failure). We should note that for the major part of the datasets collected for predicting the immunological or virological effectiveness of the failure of ART, the number of isolates was too small to build models.

## 3. Discussion

### 3.1. Predicting Drug Exposure

From the results of the prediction of drug exposure and effective/failed drug combinations, we could observe the association between nucleotide sequences encoding HIV-1 PR and a set of drugs taken by a patient with a prevalent isolate that was collected and subjected to sequencing. Although there was an association between drug exposure and drug resistance, average AUC/ROC values were about 0.81, while the standard AUC/ROC accuracy of classifying HIV variants into resistant and susceptible was above 0.90 [10]. The following reasons may explain this. First, a pretreatment state of the viral sequence is unknown. While for any new patient, a prevalent viral variant is not exposed to any drug until he/she starts antiretroviral therapy, some mutations may appear in the virus before the patient is infected with this particular viral variant. Second, the data on the patients’ adherence to treatment are unavailable. We believe that collecting these data could help to improve the quality of the data about drug exposure of sequences and, therefore, may lead to a higher accuracy of prediction when the particular score of drug adherence is considered as a parameter.

A. Pironti and coauthors [17] showed the possibility of the application of drug treatment history for antiretroviral drug optimization. Both A. Pironti and we observed a clear association between viral exposure to a drug and resistance to the same drug, which varied depending on the drug.

Summarizing our observations, an approach that allows predicting drug treatment history can help physicians to decide at an early stage, which drugs should be taken by a patient who has a prevalent viral variant that might previously has been exposed to a particular drug or drug combinations.

### 3.2. Predicting Treatment Failure and Treatment Effectiveness

The average performance for predicting the effectiveness and failure of combinations for underrepresented combinations was lower than the predictive performance for viral resistance and sequence exposure to a single drug (AUC/ROC 0.79). We suggest several possible explanations for this observation. First, the dataset for the prediction of treatment history included over 10,000 samples, whereas the dataset for the prediction of treatment failure/effectiveness was much smaller (less than 1000 samples). Second, we assumed that each drug taken by a patient might affect viral fitness. Thus, changes in HIV-1 nucleotide sequences could be more considerable after a long duration of therapy, reflected in the dataset for the prediction of drug exposure (treatment history).

A combination of drugs was considered to be failed if the number of CD4^+^ cells was below 250 cells/mm^3^ during antiretroviral therapy (immunological failure). A drug combination was associated with virological failure of ART if the viral load was above 5000 copies/mL (see also the Materials and Methods for details). There was some uncertainty in the determination of the thresholds of CD4+ cell counts in mm^3^ and the number of RNA copies in mL [26,27]. HIV resistance data are characterized by the overall low reproducibility of biological data [28,29,30,31,32]. Despite these observations, there was an association between HIV-1 nucleotide sequence and treatment failure/effectiveness. The exclusion of any one descriptor (number of CD4+ cells, RNA copies, P_1_ and P_0_ values, nucleotide descriptors) led to decrease of prediction accuracy. Therefore, all types of descriptors used for prediction are essential for the prediction of both virological and immunological therapy failure/effectiveness of therapy.

Since for some schemes of ART AUC/ROC, the accuracy of prediction is lower than 0.80, we suggest a few strategies that might be helpful to improve the prediction accuracy of HIV/AIDS treatment effectiveness/failure. First, there is a need for a bigger collection of HIV/AIDS treatment schemes along with clinical data and a score reflecting patients’ adherence to treatment. Such collections can help researchers to perform their studies on HIV/AIDS treatment effectiveness/failure taking into account clinical data and adherence of a patient to the treatment. Second, probably the comorbidities of a patient should be taken into account when a prediction of HIV/AIDS treatment effectiveness/failure is performed.

Contemporary research studies focus on the investigation of both HIV-1 and host factors to develop vaccines against HIV-1, which may represent the basis for the novel therapeutic approaches [4,5,33]. While developing new strategies of treatment can be beneficial for HIV-1 prevention and cure, antiretroviral treatment is still used worldwide, so the approaches leading to its optimization still can have an impact on the improvement of HIV-1 therapy strategies. On the other hand, since the represented approach is based on the computational analysis of HIV-1 nucleotide sequences, it can be applied for the analysis of some other HIV-1 proteins, having an impact on HIV-1 resistance to the antiretroviral therapy or playing a role in some new strategies to prevent or treat HIV/AIDS.

In summary, our computational experiments proved an association between viral genotype and treatment history of a particular viral isolate occurring in a patient or group of patients. It was possible to predict virological and immunological therapy failure/effectiveness based on the descriptors generated from a nucleotide sequence in combination with other descriptors, reflecting laboratory data and the clinical status of the patient, such as CD4^+^ count and viral load. Application of the computational models developed to predict HIV drug resistance, HIV treatment history, and virological and immunological therapy failure/effectiveness could be helpful in the optimization and personalization of antiretroviral therapy, which could be particularly important taking into account the toxicity and adverse effects [34,35,36] of drugs comprising ART schemes.

## 4. Materials and Methods

**Figure 3:**
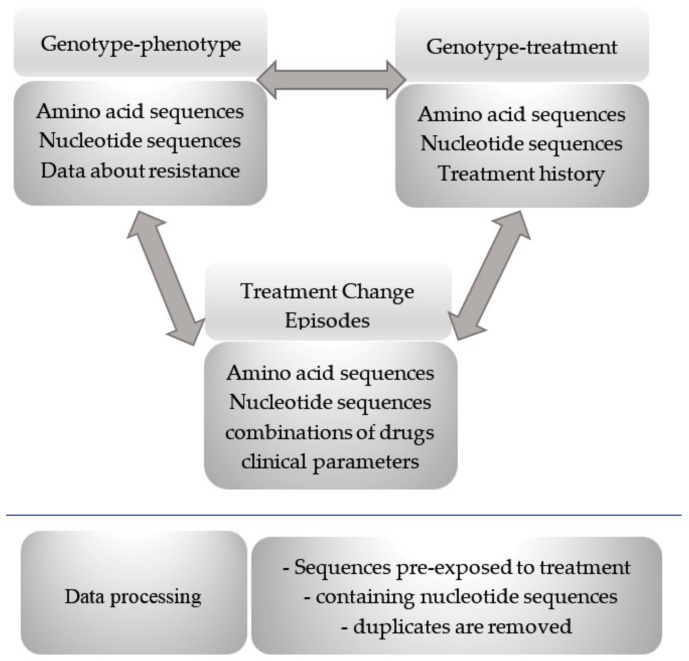
Data processing workflow to compile study datasets. The arrows indicate that this is a certain overlap between three data point types (1) Genotype-phenotype relationship, (2) Genotype-treatment relationship and (3) Treatment Change Episodes.

Datasets were compiled using information from the STDB [20], which provides three distinct data point types: genotype–phenotype (i.e., Genotype Resistance (GR)), Genotype–Treatment (GT), and Treatment Change Episodes (TCE). Details of these three data types are given in Figure 3. GT data, except for information on resistance, included treatment history for each patient, i.e., a set of all drugs ever taken by the patient from the start of any therapy. TCE data, which were provided in XML format, contained detailed information on the applied therapy, i.e., the starting point, the duration of each episode of treatment, and the set of drugs taken by the patient in the current treatment episode. These data also included information on the nucleotide sequences from the patient at a fixed time, viral load, and the number of CD4^+^ cells as an indicator of therapy effectiveness. Each file with TCE usually contained some treatment episodes distinguished by the set of drugs. TCE data did not include information on the resistance to each drug and the complete list of drugs ever taken by a patient. We processed the data of the three types mentioned above (see also Figure 3). Data processing involved three stages: (1) matching data of GR and GT types; (2) selection of only those isolates for which nucleotide sequences were available; and (3) selection of isolates pre-exposed to therapy and removal of duplicates.

### 4.1. Training Sets

Training sets were created by merging data of all three types from the STDB. The “HIV PR treatment history dataset” contained 9986 isolates with data on treatment history using PR inhibitors; the “HIV PR combinations dataset” included 852 isolates with information on drug combinations simultaneously taken by a patient. We also classified HIV PR combinations into effective and less effective based on the number of CD4+ cells and viral load obtained during therapy, according to the recommendations of the World Health Organization [25].

We suggest classification based on two class types: exposure to a particular drug set (treatment history prediction based on the HIV PR treatment history dataset) and virological and immunological treatment failure/effectiveness predicted for a particular HIV viral variant (based on the HIV PR combination dataset). We further summarized their description as types of association between sequence and clinical data.

### 4.2. Algorithm

**Figure 4:**
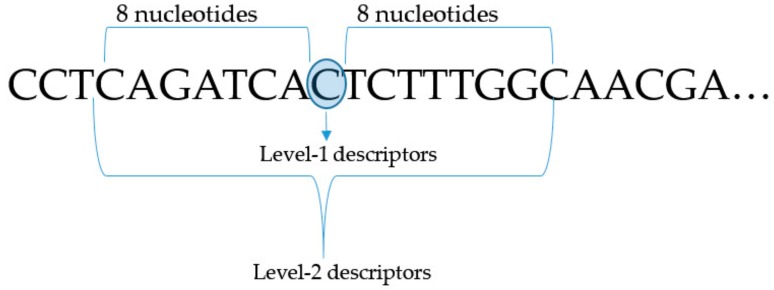
Principle of data processing to compile study datasets. The center position for creating MNA descriptors for a given example is represented in a blue circle.

In our method, we used a modified naïve Bayesian classifier implemented in the PASS program [19,21,24]. Based on our previous experience in building models of HIV-1 resistance based on nucleotide sequences, we used short nucleotides as descriptors in the PASS algorithm. We represented the nucleotide sequence of each isolate as a set of short nucleotide fragments. Short fragments were created by moving along the sequence and cutting eight nucleotides before and after the center position. Each central position was at a distance of four nucleotides from the previous center. The nucleotide in the current central position and nucleotides before and after that position comprised the descriptor of the Multilevel Neighborhoods of the Nucleotide (MNN). Therefore, each first level MNN descriptor corresponded to the fragment of 9 nucleotides; each second level MNN descriptor corresponded to the fragment of 17 nucleotides, and so on. In this study, we used the MNN descriptors of the first and second levels (Figure 4). Second level descriptors were stored in the knowledge base during the training procedure with data about a particular sample belonging to a specific class.

The prediction algorithm was described earlier in detail in the application to amino acid descriptors [19]. In our approach, we did not use amino acid sequences, so we modified the algorithm to be applied to nucleotide sequences. We estimated the probability P_1_ and P_0_ of the isolate to belong and not belong to class C, associated with clinical data. The details of the algorithm are given in the Supplementary Materials (the section “PASS Algorithm for Nucleotide Sequences”).

The random forest (RF) approach was applied in combination with the PASS approach, as described below. The binary descriptors for the random forest classifier were obtained based on nucleotide sequences, as described in [10]. We generated the set of short nucleotide fragments (descriptors). The length of each short fragment was 16 nucleotides. In total, over 1100 nucleotide descriptors were generated. Next, we selected 305 descriptors with the frequency of occurrence over 10. For each nucleotide sequence, we designed a set of 305 binary descriptors, where “0” was added to the set if the descriptor of the considered sequence could not be found in the entire set of 305 descriptors and “1” if the descriptor was found in the set. The P_1_ and P_0_ values calculated by PASS, the number of CD4+ cells, and the logarithmic value of viral RNA copies were added to the set of binary descriptors. The random forest classifier implemented in Weka 3.8.4 was used for building models.

## 5. Conclusions

We presented an application of the PASS approach to the prediction of the treatment history of patients with HIV/AIDS based on nucleotide sequences of the HIV-1 isolate. The average AUC/ROC prediction accuracy was 0.81 (±0.07). We also demonstrated the combined application of PASS and random forest classifiers for the prediction of immunological and virological effectiveness/failure of antiretroviral therapy. The average AUC/ROC accuracy of this kind of prediction was 0.84 (±0.07). Prediction results of treatment history and effective/failed combinations based on computational methods could be helpful in HIV-1 therapy optimization.

## Figures and Tables

**Table 1:** Results of the classification of Human Immunodeficiency Virus Type 1 (HIV-1) Protease (PR) sequences according to exposure to HIV-1 PR Inhibitors.

Drug Set ^1^	Sample Number	Period of Exposure ^2^		AUC/ROC ^3^	AUC/ROC_20_^3^
LPV ^4^	2896	63 (57)		0.94	0.91
NFV	1334	68 (62)		0.81	0.80
IDV	984	74 (72)		0.77	0.79
IDV, NFV, RTV, SQV	425	160 (77)		0.83	0.82
IDV, NFV	396	160 (81)		0.81	0.78
IDV, NFV, SQV	238	127 (71)		0.80	0.79
RTV, TPV	132	N/A ^5^		0.91	0.90
APV, IDV, NFV, RTV, SQV	121	218 (74)		0.86	0.84
ATV	106	39 (22)		0.81	0.80
IDV, LPV	91	129 (101)		0.81	0.80
APV	66	41 (29)		0.82	0.80
IDV, LPV, NFV, RTV, SQV	70	272 (107)		0.77	0.76
LPV, RTV	35	182 (104)		0.81	0.80
RTV, SQV	35	91 (60)		0.81	0.79
Other (average)	3314	N/A ^5^		0.79	0.76
**Total**	**10,243**		**0.81**		**0.80**

^1^ ATV, Atazanavir; APV, Amprenavir; DRV, Darunavir; FPV, Fosamprenavir; IDV, Indinavir; LPV, Lopinavir; NFV, Nelfinavir; RTV, Ritonavir, SQV, Saquinavir; TPV, Tipranavir; ^2^ period of drug exposure: weeks, average (standard deviation) ^3^ AUC/ROC: area under the ROC curve obtained in leave-one-out cross-validation (LOO CV); AUC/ROC_20_, area under the ROC curve obtained in fivefold CV; ^4^ HIV-1 PR inhibitors were taken in combination with other antiretroviral drug(s); ^5^ N/A data are not available.

**Table 2:** Prediction results of immunological effectiveness/failure of treatment for HIV-1 protease inhibitors obtained using the random forest classifier based on the features of nucleotide sequences of a particular viral variant and clinical parameters (CD4^+^ cells and the number of viral RNA copies).

Drug Combinations	Sequence Number	AUC/ROC	AUC/ROC_20_
No PR inhibitor, effective	234	0.94	0.91
NFV ^1^, effective	147	0.90	0.86
LPV ^1^, effective	58	0.77	0.79
RTV ^1^, APV ^1^, effective	26	0.82	0.80
IDV ^1^, effective	28	0.91	0.90
No PR inhibitor, failed	42	0.94	0.92
SQV ^1^, RTV ^1^, failed	26	0.94	0.92
NFV ^1^, failed	23	0.90	0.89
Other (rare combinations)	268	0.79	0.76
**Average**	**852**	**0.84**	**0.82**

^1^ HIV-1 PR inhibitors were typically taken in combination with other antiretroviral drugs (Reverse Transcriptase (RT) inhibitors).

## Supplementary Materials

Supplementary Materials can be found at https://www.mdpi.com/1422-0067/21/3/748/s1.

## Abbreviations

HIV-1	Human Immunodeficiency Virus Type 1
ART	Antiretroviral Therapy
RT	Reverse Transcriptase
PR	Protease
WHO	The World Health Organization
PASS	Prediction of Activity Spectra for Substances
RF	Random Forest
STDB	The Stanford HIV Resistance Database
ROC	Receiver Operating Characteristic
AUC/ROC	The Area under ROC Curve
ATV	Atazanavir
APV	Amprenavir
DRV	Darunavir
FPV	Fosamprenavir
DRV	Darunavir
IDV	Indinavir
LPV	Lopinavir
NFV	Nelfinavir
RTV	Ritonavir
SQV	Saquinavir
TPV	Tipranavir
