# Supplementary material for: A Computational Approach for the Prediction of Treatment History and the Effectiveness or Failure of Antiretroviral Therapy
Source: Int J Mol Sci. 2020 Jan 23;21(3):748. doi: 10.3390/ijms21030748 (PMC7037494; doi:10.3390/ijms21030748)
Supplement: Supplementary file 1 [file ijms-21-00748-s001.pdf]

Table S1. The distribution of the mutations, most frequently occurred in the data sets of isolates associated with immunologically effective and failed drugs and drugs combinations of HIV-1 protease inhibitors.

Drug or drug combination	LPV (lopinavir)				
**Position**	**Changes of amino acids residues**	**All**	**Resistant**	**Immunologically effective**	**Immunologically failed**
30	N	5.38	5.9	5.1	3
32	I	8.51	12	5.05	10
47	V	5.33	7.03	9.26	7
48	V	3.54	7.03	5.36	4
48	M	2	1	0	0
50	V	1.79	2.9	2	0
54	V	20	33	28	19
76	V	2.3	4	3.5	4
82	A	19	32	23	20
84	V	16	26	19	14
88	D	9.9	6	8.9	4
**Drug or drug combination**	**NFV (nelfinavir)**				
**Position**	**Changes of amino acids residues**	**All**	**Resistant**	**Immunologically effective**	**Immunologically failed**
30	N	5.38	10	18.6	20
32	I	8.51	14.6	6.78	6
47	V	5.33	7.6	6.3	4
48	V	3.54	6	0	0
48	M	2	1.1	0	0
50	V	1.79	2.5	0	0
54	V	20	35	6.44	4
76	V	2.3	4.1	2.5	0
82	A	19	34	23	27
84	V	16	28	19	17
88	D	9.9	9.4	2	0
**Drug or drug combination**	**IDV (indinavir)**				
**Position**	**Changes of amino acids residues**	**All**	**Resistant**	**Immunologically effective**	**Immunologically failed**
30	N	5.38	1.7	0.5	0
32	I	8.51	1.9	4	12
47	V	5.33	1.9	6	11
48	V	3.54	6	5	6
48	M	2	4.9	0	0
50	V	1.79	0	0	0
54	V	20	35	8	16
76	V	2.3	4.1	0	0
82	A	19	41.6	4	27
84	V	16	13	2	0
88	D	9.9	2.2	0	0

Drug or drug combination	RTV (ritonavir), APV (amprenavir)				
**Position**	**Changes of amino acids residues**	**All**	**Resistant**	**Immunologically effective**	**Immunologically failed**
30	N	5.38	4.2	5	N/A
32	I	8.51	9.5	11	N/A
47	V	5.33	7.6	6.3	N/A
48	V	3.54	6	8.4	N/A
48	M	2	8.4	53	N/A
50	V	1.79	2.5	0	N/A
54	V	20	35	25	N/A
76	V	2.3	4.1	0	N/A
82	A	19	41.6	30	N/A
84	V	16	28	19	N/A
88	D	9.9	5.7	30	N/A

Drug or drug combination	SQV (saquinavir), RTV (ritonavir)				
**Position**	**Changes of amino acids residues**	**All**	**Resistant**	**Immunologically effective**	**Immunologically failed**
30	N	5.38	2.1	11	13
32	I	8.51	6.1	4	0
47	V	5.33	2.4	0	0
48	V	3.54	10.7	18	15
48	M	2	0	0	4
50	V	1.79	1.8	0	0
54	V	20	34	41	53
76	V	2.3	31.7	0	14
82	A	19	33.1	15	20
84	V	16	28.5	8	13
88	D	9.9	2.6	24	20

Table S2. The distribution of the mutations, most frequently occurred in the data sets of isolates associated with virologically effective and failed drugs and drugs combinations of HIV-1 protease inhibitors.

Drug or drug combination	LPV (lopinavir)				
**Position**	**Changes of amino acids residues**	**ALL**	**Resistant**	**Virologically effective**	**Virologically failed**
30	N	5.38	5.9	6	4
32	I	8.51	12	9	6
47	V	5.33	7.03	8.5	10
48	V	3.54	7.03	6.06	8
48	M	2	1	0	0
50	V	1.79	2.9	0	0
54	V	20	33	8	0.3
76	V	2.3	4	4.5	5
82	A	19	32	15	19
84	V	16	26	13	10
88	D	9.9	6	6.9	4

Drug or drug combination	NFV (nelfinavir)				
**Position**	**Changes of amino acids residues**	**ALL**	**Resistant**	**Virologically effective**	**Virologically failed**
30	N	5.38	10	33	25
32	I	8.51	14.6	1.6	6
47	V	5.33	7.6	0	4
48	V	3.54	6	0	0
48	M	2	1.1	0	0
50	V	1.79	2.5	0	0
54	V	20	35	6	4
76	V	2.3	4.1	2.5	1
82	A	19	34	13	21
84	V	16	28	6.5	8
88	D	9.9	9.4	8	0

Drug or drug combination	SQV (saquinavir), RTV (ritonavir)				
**Position**	**Changes of amino acids residues**	**ALL**	**Resistant**	**Virologically effective**	**Virologically failed**
30	N	5.38	1.7	9.8	24
32	I	8.51	1.9	8	10
47	V	5.33	1.9	1.7	2.8
48	V	3.54	6	7	9
48	M	2	4.9	0	0.5
50	V	1.79	0	0	0
54	V	20	35	26	34
76	V	2.3	4.1	4	21
82	A	19	41.6	14	21
84	V	16	13	0	0
88	D	9.9	2.2	6	20.5

Results of prediction of virological effectiveness for HIV-1 protease inhibitors.

Table S3. Prediction results of virological effectiveness/failure of treatment based on both the features of nucleotide sequence of particular viral variant and clinical parameters (CD4+ cells and number of viral RNA copies).

Drug Combinations	Sample Number	AUC/ROC	AUC/ROC_{20}
No PR inhibitor[1], effective	62	0.94	0.93
NFV, effective[1]	42	0.94	0.92
None of protease inhibitor[1], failed	212	0.88	0.84
NFV, failed[1]	125	0.83	0.83
LPV, failed[1]	64	0.76	0.71
SQV, RTV, failed[1]	42	0.84	0.90
Other (rare combinations) [1]	305	0.78	0.76
**Average**	**852**	**0.82**	**0.81**

[1] HIV-1 protease inhibitors were typically taken in combination with other antiretroviral drugs (reverse transcriptase inhibitors).

Results of prediction of sequence exposure to the drug(s) and virological/immunological effectiveness or failure of therapy for HIV-1 reverse transcriptase inhibitors.

Table S4. The AUC/ROC values of classifying exposure of the sequence to the drug or drug combinations of HIV-1 reverse transcriptase inhibitors.

Activity Type	Number	AUC/ROC
3TC,AZT,D4T,DDI	176	0.69
3TC,AZT	160	0.67
AZT	158	0.82
3TC,AZT,D4T,DDC,DDI	104	0.71
3TC,AZT,D4T,DDI,NVP	104	0.72
AZT,DDI	86	0.83
3TC,AZT,D4T	82	0.68
D4T,DDI	81	0.69
3TC,D4T	79	0.68
3TC,AZT,D4T,DDC	74	0.69
3TC,AZT,D4T,DDC,DDI,NVP	60	0.65
3TC,AZT,D4T,DDI,EFV	45	0.69
3TC,AZT,DDI	41	0.68
AZT,DDC	34	0.78
3TC,AZT,D4T,NVP	30	0.69
3TC,AZT,DDC	29	0.74
3TC,AZT,D4T,DDC,NVP	19	0.85
3TC,ABC,AZT,D4T,DDI,NVP	18	0.72
DDI	15	0.91
DLV	15	0.97
3TC,AZT,D4T,DDC,DDI,DLV	14	0.73
3TC,AZT,D4T,DDC,DDI	12	0.83

3TC,AZT,D4T,DDC,DDI,EFV,NVP	12	0.78
3TC,AZT	11	0.71
3TC,ABC,AZT,D4T	10	0.94
AZT,DLV	9	0.89
3TC,AZT	9	0.84
3TC,AZT,D4T,DDI,EFV,NVP	9	0.75
3TC,D4T,DDI	8	0.71
3TC,ABC,AZT,D4T,DDI,EFV	8	0.85
3TC,ABC,AZT,D4T,DDC,DDI,NVP	7	0.74
3TC,ABC,D4T,NRTI,NVP	7	1.00
3TC,AZT,D4T,EFV	7	0.77
3TC,ABC,AZT,NRTI,NVP	6	1.00
3TC,ABC,AZT,D4T,DDI,EFV,NVP	6	0.75
3TC,AZT,D4T,DDI,EFV,NVP	5	1.00
3TC,AZT,D4T,DDI,DLV,NVP	5	0.82
3TC,ABC,DDI,NRTI,NVP	5	1.00
3TC,ABC,AZT,D4T,DDC,DDI,EFV	5	0.77
3TC,ADV,AZT,D4T,DDC,DDI,EFV	4	1.00
3TC,ADV,AZT,D4T,DDI,EFV,NVP	4	0.88
3TC,ABC,D4T,EFV	4	1.00
NVP	4	1.00
3TC,D4T,EFV	4	0.89
D4T,DDI,NVP	4	0.79
3TC,ABC,AZT,D4T,DDC,DDI,NVP	3	0.89
3TC,AZT,D4T,DDC,DDI,EFV,NVP	3	0.84
3TC,ADV,AZT,DDI,EFV,NVP	3	1.00
3TC,ABC,AZT,NVP	3	1.00
3TC,ABC,DDC,DDI	3	1.00
3TC,ABC,D4T	3	1.00
3TC,ABC,AZT,D4T	3	1.00
**Average**		0.83

Table S5 The AUC/ROC values of classifying immunological effectiveness/failure of therapy for HIV-1 reverse transcriptase inhibitors.

No	Activity Type	Number	AUC/ROC	AUC/ROC_{20}
1	TDF, FTC, effective[1]	10	0.70	0.70
3	ABC, D4T, effective[1]	6	0.71	0.70

[1]HIV-1 reverse transcriptase inhibitors were typically taken in combination with other antiretroviral drugs (protease, integrase inhibitors).

Table S6. The AUC/ROC values of classifying virological effectiveness/failure of therapy for HIV-1 reverse transcriptase inhibitors.

No	Activity Type	Number	AUC/ROC	AUC/ROC_{20}
1	AZT,3TC_failed[1]	5	0.79	0.74
3	D4T, DDI_effective[1]	9	0.86	0.82
4	ABC,D4T_effective[1]	10	0.78	0.79

[1]HIV-1 reverse transcriptase inhibitors were typically taken in combination with other antiretroviral drugs (protease, integrase inhibitors).

Algorithm of PASS for classifying nucleotide sequences according to drug exposure and prediction of viral/immunological effectiveness or failure

Probability calculation was based on the estimation of (i) a priori probability $P(C_k)$ to find the nucleotide sequence from a certain class k, and (ii) conditional probability $P(C_k/D_i)$ of the nucleotide sequence to belong to the certain class k if the sequence contained descriptor D_i. Frequency estimation of probabilities $P(C_k)$ and $P(C_k/D_i)$ depended on the following metrics (Equations 1 and 2):

$$P(C_k) = \frac{N_k}{N} \quad (1)$$

$$P(C_k/D_i) = \frac{N_{ik}}{N_i}, \quad (2)$$

where N is the total number of nucleotide sequences in the training set, N_k is the number of nucleotide sequences belonging to class k, N_{ik} is the number of sequences belonging to class k, and containing descriptor D_i. The probabilities $P(C_k)$ and $P(C_k/D_i)$ were calculated, and then B_k was calculated (Equations 3–5).

$$S_{0k} = 2P(C_k) - 1 \quad (3)$$

$$S_k = sin\left[\frac{1}{m}\sum arcsin(2P(C_k/D_i) - 1)\right] \quad (4)$$

$$B_k = \frac{S_k - S_{0k}}{1 - S_k S_{0k}}. \quad (5)$$

For class k, $B_k = 1$ if for all descriptors $P(C_k/D_i) = 1$. Alternatively, $B_k = -1$ if $P(C_k/D_i) = 0$ for all descriptors. During the training procedure, distribution functions F_{k1} and F_{k0} were calculated on the basis of the values of B_{k1} and B_{k0} obtained for the sets of sequences that belonged and did not belong to a certain class C. Probabilities P_{k1} and P_{k0} for each input nucleotide sequence were based on calculated estimates using distribution functions F_{k1} and F_{k0}.
